# Comprehensive Analysis of circRNA-miRNA-mRNA Regulatory Network and Novel Potential Biomarkers in Acute Myocardial Infarction

**DOI:** 10.3389/fcvm.2022.850991

**Published:** 2022-07-07

**Authors:** Jiahe Wu, Chenze Li, Zhe Lei, Huanhuan Cai, Yushuang Hu, Yanfang Zhu, Tong Zhang, Haoyan Zhu, Jianlei Cao, Xiaorong Hu

**Affiliations:** ^1^Department of Cardiology, Zhongnan Hospital of Wuhan University, Wuhan, China; ^2^Institute of Myocardial Injury and Repair, Wuhan University, Wuhan, China

**Keywords:** acute myocardial infarction, circRNA, bioinformatics analysis, biomarker, circRNA-miRNA-mRNA regulatory network

## Abstract

**Background:**

Circular RNA (circRNA) plays an important role in the regulation of gene expression and the occurrence of human diseases. However, studies on the role of circRNA in acute myocardial infarction (AMI) are limited. This study was performed to explore novel circRNA-related regulatory networks in AMI, aiming to better understand the molecular mechanism of circRNAs involvement in AMI and provide basis for further scientific research and clinical decision-making.

**Methods:**

The AMI-related microarray datasets GSE160717 (circRNA), GSE31568 (miRNA), GSE61741 (miRNA), and GSE24519 (mRNA) were obtained from the Gene Expression Omnibus (GEO) database. After differential expression analysis, the regulatory relationships between these DERNAs were identified by online databases circBank, circInteractome, miRDB, miRWalk, Targetscan, and then two circRNA-miRNA-mRNA regulatory networks were constructed. Differentially expressed genes (DEGs) in this network were selected followed by enrichment analysis and protein–protein interaction (PPI) analysis. Hub genes were identified using Cytohubba plug-in of Cytoscape software. Hub genes and hub gene-related miRNAs were used for receiver operating characteristic curve (ROC) analysis to identify potential biomarkers. The relative expression levels of these biomarkers were further assessed by GSE31568 (miRNA) and GSE66360 (mRNA). Finally, on the basis of the above analysis, myocardial hypoxia model was constructed to verify the expression of Hub genes and related circRNAs.

**Results:**

A total of 83 DEcircRNAs, 109 CoDEmiRNAs and 1204 DEGs were significantly differentially expressed in these datasets. The up-regulated circRNAs and down-regulated circRNAs were used to construct a circRNA-miRNA-mRNA regulatory network respectively. These circRNA-related DEGs were mainly enriched in the terms of “FOXO signaling pathway,” “T cell receptor signaling pathway,” “MAPK signaling pathway,” “Insulin resistance,” “cAMP signaling pathway,” and “mTOR signaling pathway.” The top 10 hub genes ATP2B2, KCNA1, GRIN2A, SCN2B, GPM6A, CACNA1E, HDAC2, SRSF1, ANK2, and HNRNPA2B1 were identified from the PPI network. Hub genes GPM6A, SRSF1, ANK2 and hub gene-related circRNAs hsa_circ_0023461, hsa_circ_0004561, hsa_circ_0001147, hsa_circ_0004771, hsa_circ_0061276, and hsa_circ_0045519 were identified as potential biomarkers in AMI.

**Conclusion:**

In this study, the potential circRNAs associated with AMI were identified and two circRNA-miRNA-mRNA regulatory networks were constructed. This study explored the mechanism of circRNA involvement in AMI and provided new clues for the selection of new diagnostic markers and therapeutic targets for AMI.

## Introduction

Cardiovascular disease is a serious threat to human health. Ischemic heart disease remains one of the leading causes of death and disability worldwide (1). The high incidence and mortality of acute myocardial infarction (AMI) causes a serious social and health economic burden and affects the quality of human life.

Acute myocardial infarction is the result of myocardial ischemic necrosis caused by acute interruption of myocardial blood flow, often characterized by acute thrombosis superimposed on atherosclerotic plaque rupture (2). Early successful reperfusion therapy [thrombolytic therapy or percutaneous coronary intervention (PCI)] is the best way to reduce the size of myocardial infarction and improve the clinical outcome (3). Although the detection of higher sensitivity troponin improved the recognition of AMI patients suitable for PCI, it still had some defects in the recognition of early infarction and mild cardiac injury (4). Recently, non-coding RNAs (ncRNA), including circular RNAs (circRNA), long non-coding RNAs (lncRNA), microRNAs (miRNAs), as new biological markers of AMI, has attracted the attention of scientists.

Circular RNAs, characterized by a covalently closed continuous loop, is a type of circular single-stranded RNA different from other linear RNAs (5). They are produced by reverse splicing of the precursor mRNA transcript, in which the upstream splicing receptor connects to the downstream splicing donor (6). As an effective sponge of miRNA, circRNA interacts with miRNA to regulate mRNA expression (7). A large number of studies have demonstrated that circRNA is involved in the development of a variety of cardiovascular diseases, including heart failure (8), atrial fibrillation (9), diabetic cardiomyopathy (10), dilated cardiomyopathy (11), acute myocardial infarction (12), and myocardial ischemia-reperfusion injury (13). A study reported that circRNA SNRK acts as a sponge for miR-103-3p and regulates myocardial apoptosis through GSK3β/β-catenin pathway. Overexpression of circRNA SNRK can significantly reduce myocardial cell apoptosis and promote cardiac repair in myocardial infarction rats (14). Another study showed that circRNA Ube3a derived from M2 macrophages can promote proliferation, migration and phenotypic transformation of cardiac fibroblasts (CFs) cells by targeting miR-138-5p/RHOC axis, aggravating cardiac fibrosis after AMI (15). In addition, circ_0023461 silencing protects cardiomyocytes from hypoxia-induced dysfunction by targeting miR-370-3p/PDE4D axis (16). These findings suggest that circRNA is involved in the pathophysiological process of AMI and plays an important regulatory role in the injury and repair of infarcted myocardium. Therefore, circRNA may be used as a biomarker of myocardial infarction.

**FIGURE 1:**
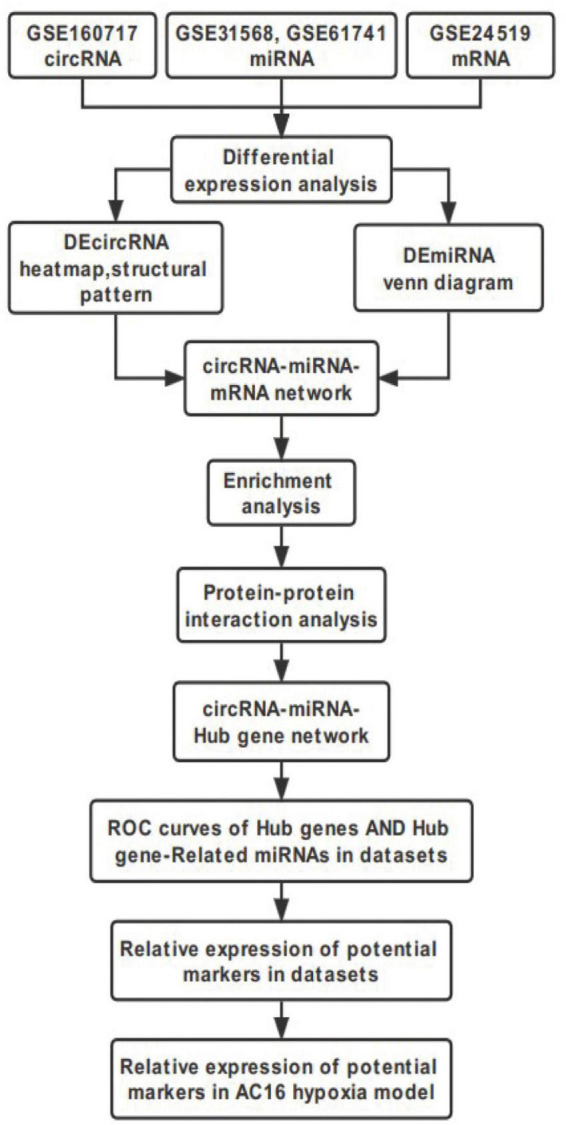
Flowchart of the steps performed in this study.

This study was performed to construct the circRNA-miRNA-mRNA regulatory Network and identify novel potential biomarkers in AMI. Datasets GSE160717, GSE31568, GSE61741, GSE24519, and GSE66360 were downloaded and then analyzed using Bioinformatics analysis. Our study would provide new insights of molecular mechanisms of circRNAs involved AMI, aiming to understand the development and progression of AMI and find targets for its diagnosis and treatment. The workflow of the specific analysis is shown in Figure 1.

## Materials and Methods

### Data Resource

The microarray datasets GSE160717 (circRNA), GSE31568 (miRNA), GSE61741 (miRNA), GSE24519 (mRNA), and GSE66360 (mRNA) were obtained from the Gene Expression Omnibus (GEO) database.^1^ GSE160717 is based on the GPL21825 platform (074301 Arraystar Human CircRNA microarray V2). GSE31568 and GSE61741 are based on the GPL9040 platform (febit Homo Sapiens miRBase 13.0). GSE24519 is based on the GPL2895 platform (GE Healthcare/Amersham Biosciences CodeLink Human Whole Genome Bioarray). GSE66360 is based on the GPL2895 platform ([HG-U133_Plus_2] Affymetrix Human Genome U133 Plus 2.0 Array). These datasets satisfy the following characteristics: (1) Organism: Homo sapiens; (2) Sample source: Blood; and (3) Grouping: AMI group and healthy controls. Table 1 shows the detailed information of these five microarray datasets. These datasets are widely used for bioinformatics analyses to explore the molecular mechanisms associated with AMI (17–19).

**TABLE 1:** The detailed information of the 5 microarray datasets.

Data source	Organism	Platform	Year	Sample source	Sample size (AMI/CON)	Detected RNA type
GSE160717	Homo sapiens	GPL21825	2020	Blood	3/3	circRNA
GSE31568	Homo sapiens	GPL9040	2011	Blood	20/70	miRNA
GSE61741	Homo sapiens	GPL9040	2014	Blood	62/94	miRNA
GSE24519	Homo sapiens	GPL2895	2017	Blood	17/4	mRNA
GSE66360	Homo sapiens	GPL570	2015	Blood	49/50	mRNA

### Differential Expression Analysis

Raw sequencing data of GSE160717 (circRNA), GSE31568 (miRNA), GSE61741 (miRNA), and GSE24519 (mRNA) were downloaded from the abovementioned platform and preliminary differential expression analysis between AMI samples and control was performed using GEO2R.^2^ Benjamini-hochberg method was used to correct adj. P for potential false positive results. FDR < 0.05 and |logFC| > 1.0 was set as the cut-off criteria of DERNAs. CoDEmiRNAs in GSE31568 and GSE61741were identified using Venn diagram web tool.^3^ Based on relative expression information in raw sequencing data, the heatmap of DEcircRNAs were produced using the package gg plots of R software (version: x64 3.2.1) (20).

### circRNA and miRNA ID Conversion

Using circBase database,^4^ the circRNA name in GSE160717 containing six digits was converted into circBase ID containing seven digits. circRNAs that do not match in the annotation file were further blatted to complete the ID conversion. The miRDB^5^ database was used to convert the miRNA ID in GSE31568 and GSE61741 from the previous version to the latest version.

### DEcircRNA Annotation and Structure Prediction

CircBase is a circRNA-related database that records circRNA-related annotation information, such as sequence, gene and genomic location (21). DEcircRNAs in GSE160717 were entered into the circBase database to get their annotation information. CSCD^6^ database is the first comprehensive cancer-specific circRNA database which can predict the microRNA response element (MRE) sites, RNA binding protein (RBP) sites, and potential open reading frames (ORF) (22). Structure of these DEcircRNAs were predicted and visualized using CSCD.

### Construction of the circRNA-miRNA-mRNA Regulatory Network

Based on DEcircRNAs and CoDEmiRNAs, the circRNA-miRNA interactions were predicted using website tools of circBank^7^ and circInteractome^8^ (23, 24). Based on CoDEmiRNAs and differentially expressed genes (DEGs), the miRNA–mRNA interactions were predicted using website tools of miRDB,^9^ Targetscan (version7.2^10^), and miRWalk^11^ (25–28). According to these regulatory relationships, the up-regulated circRNAs and down-regulated circRNAs were used to construct a circRNA-miRNA-mRNA regulatory network respectively. The network diagram wsa visualized by Cytoscape software (version 3.7.1).

### Functional and Pathway Enrichment Analysis

The Database for Annotation, Visualization, and Integrated Discovery (DAVID; version6.8^12^) was used to perform Gene Ontology (GO) analysis and Kyoto Encyclopedia of Genes and Genomes (KEGG) pathway analysis of DEGs in these two circRNA-miRNA-mRNA regulatory networks, respectively (29). *P* value < 0.05 was set as the threshold to identify statistically significant pathways.

### Construction of the Protein–Protein Interaction Network and Module Analysis

In order to better understand the molecular mechanism of circRNA’s involvement in transcriptional regulation, a protein–protein interaction network (PPI network) of DEGs in the circRNA-miRNA-mRNA network was constructed. The String database (version10.0^13^) was used to predict the interaction between proteins, and a PPI network was established by Cytoscape software (version 3.7.1) subsequently.

The scores of Maximal Clique Centrality (MCC) algorithm was set as the criteria (30, 31), and the top10 Hub genes with high connectivity in the PPI network was screened out using the Cytohubba plug-in of Cytoscape software. In addition, the MCODE plug-in of Cytoscape software was used to predict meaningful modules with default parameters as thresholds. Finally, the key pathways of these modules were predicted by KOBAS (version 3.0^14^) database.

### Construction of the circRNA-miRNA-Hub Gene Regulatory Network

Based on hub genes identified in PPI network, corresponding miRNAs and circRNAs were selected from circRNA-miRNA-mRNA regulatory networks mentioned above. R software ggplot2, ggalluvial, and dplyr package was used to draw sankey diagrams (32). The heat map of circRNAs in this sankey diagram were produced using the package gg plots of R software (version: x64 3.2.1).

### Identification and Validation of Potential Biomarkers

Hub gene and hub gene-related miRNAs in the sankey diagram were considered as candidate biomarkers for AMI. Based on their raw expression data in datasets GSE31568 (miRNA), GSE61741 (miRNA), GSE24519 (mRNA), and GSE66360 (mRNA), Graphpad Prism (version:8.0) software was used to plot the receiver operator characteristic curves and calculate the area under the curve (AUC) respectively to evaluate the predictive ability of these biomarkers for AMI. Meanwhile, hub genes and miRNAs with AUC ≥ 7.0 and *P* < 0.05 were screened as potential biomarkers. Their relative expression levels in these datasets were further evaluated by box plots. Graphpad Prism 9 software was used for analysis and mapping to compare the control and AMI groups. Comparison between the two groups was performed by independent sample T test. *P* < 0.05 was considered statistically significant.

### Cell Culture and Treatments

Human cardiomyocyte AC16 cell line were purchased from BeNa Culture Collection (Beijing, China). The cells were cultured in Dulbecco’s modified Eagle’s medium (DMEM; Gibco) with 10% fetal bovine serum (FBS, Gibco) and 1% penicillin-streptomycin (Sigma, St. Louis, MO, United States) at 37°C with 5% CO_2_.

The AC16 cardiomyocytes were exposed to 24 h of hypoxia (1% O_2_, 5% CO_2_, and 94% N_2_) in DMEM to generate a hypoxia model. AC16 cells under normoxia condition all the time were regarded as the control group.

### Real-Time Quantitative Polymerase Chain Reaction

According to the manufacturer’s protocol, total RNA was isolated using FastPure^®^ Cell/Tissue Total RNA Isolation Kit V2 (Vazyme, Nanjing, China), and the mRNA and circRNA cDNAs were synthesized with Hifair^®^ II 1st Strand cDNA Synthesis Kit (gDNA digester plus) (Ye Sen, Shanghai, China). The qRT-PCR reactions were carried out in the Bio-Rad CFX96 Real-time PCR Detection System, using Hieff UNICON^®^ Universal Blue qPCR SYBR Green Master Mix (Ye Sen, Shanghai, China). GAPDH was used as the reference genes and the relative fold change was evaluated by the method of 2^–ΔΔCt^. All primers were presented in Supplementary File 3. Comparison between the two groups was performed by independent sample T test. *P* < 0.05 was considered statistically significant.

## Results

### Identification of Differentially Expressed Genes in Acute Myocardial Infarction

**FIGURE 2:**
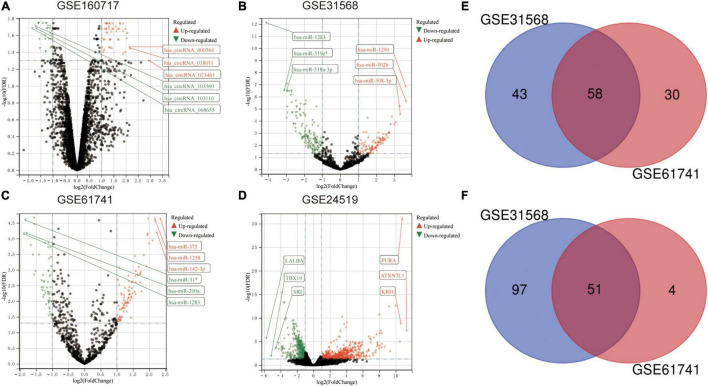
Differential expression analysis. **(A)** The volcano plot of GSE160717. **(B)** The volcano plot of GSE31568. **(C)** The volcano plot of GSE61741. **(D)** The volcano plot of GSE24519. For panels **(A–D)**, differentially expressed molecules were screened under the cut-off criteria | log2FC| > 1 and the FDR < 0.05. **(E)** Venn diagram of up-regulated DEmiRNAs in GSE31568 and GSE61741. **(F)** Venn diagram of down-regulated DEmiRNAs in GSE31568 and GSE61741.

The microarray datasets GSE160717 (circRNA), GSE31568 (miRNA), GSE61741 (miRNA), and GSE24519 (mRNA) were obtained from the Gene Expression Omnibus (GEO) database. Based on GEO2R software analysis and screening criteria mentioned above, DERNAs in each dataset were identified. There were 83 DEcircRNAs, including 50 upregulated and 33 downregulated, in GSE160717. There were 249 DEmiRNAs, including 101 upregulated and 148 downregulated, in GSE31568. There were 143 DEmiRNAs, including 88 upregulated and 55 downregulated, in GSE61741. Finally, there were 1204 DEGs, including 766 upregulated and 438 downregulated, in GSE24519. Supplementary File 1 presents detailed results of differential expression analysis. The volcano plots of these DERNAs in each dataset are shown in Figures 2A–D. The online venn tool was used to identify CoDEmiRNAs in GSE31568 and GSE61741. Finally, 58 upregulated and 51 downregulated CoDEmiRNAs were found (Figures 2E,F). The top 5 high-expressed DERNAs and the top 5 low-expressed DERNAs in each dataset are shown in Table 2.

**TABLE 2:** The top 5 high-expressed DERNAs and the top 5 low-expressed DERNAs in each dataset.

Dataset	Type	DERNA	Expression	logFC	FDR
GSE160717	circRNA	hsa_circRNA_023461	Up	2.94	4.96E-02
GSE160717	circRNA	hsa_circRNA_038011	Up	2.18	3.56E-02
GSE160717	circRNA	hsa_circRNA_400564	Up	2.15	3.44E-02
GSE160717	circRNA	hsa_circRNA_400027	Up	2.06	2.32E-02
GSE160717	circRNA	hsa_circRNA_400101	Up	2.03	2.68E-02
GSE160717	circRNA	hsa_circRNA_068655	Down	–1.84	2.08E-02
GSE160717	circRNA	hsa_circRNA_103560	Down	–1.71	1.95E-02
GSE160717	circRNA	hsa_circRNA_103110	Down	–1.67	2.08E-02
GSE160717	circRNA	hsa_circRNA_015677	Down	–1.59	1.79E-02
GSE160717	circRNA	hsa_circRNA_103111	Down	–1.59	1.80E-02
GSE31568	miRNA	hsa-miR-302b	Up	3.64	2.19E-06
GSE31568	miRNA	hsa-miR-1290	Up	3.62	1.34E-07
GSE31568	miRNA	hsa-miR-508-3p	Up	3.32	2.58E-05
GSE31568	miRNA	hsa-miR-126*	Up	3.29	5.43E-06
GSE31568	miRNA	hsa-miR-1258	Up	3.07	1.22E-04
GSE31568	miRNA	hsa-miR-1283	Down	–4.10	8.15E-13
GSE31568	miRNA	hsa-miR-519e*	Down	–3.11	3.21E-07
GSE31568	miRNA	hsa-miR-518a-3p	Down	–2.97	3.21E-07
GSE31568	miRNA	hsa-miR-1201	Down	–2.97	5.84E-05
GSE31568	miRNA	hsa-miR-31*	Down	–2.95	8.67E-08
GSE61741	miRNA	hsa-miR-375	Up	2.35	2.25E-05
GSE61741	miRNA	hsa-miR-142-3p	Up	2.23	6.26E-05
GSE61741	miRNA	hsa-miR-1258	Up	2.19	2.59E-05
GSE61741	miRNA	hsa-miR-29c*	Up	2.18	2.25E-05
GSE61741	miRNA	hsa-miR-302b	Up	2.10	1.12E-04
GSE61741	miRNA	hsa-miR-1283	Down	–1.88	6.99E-05
GSE61741	miRNA	hsa-miR-31*	Down	–1.84	2.59E-05
GSE61741	miRNA	hsa-miR-200a	Down	–1.80	6.79E-05
GSE61741	miRNA	hsa-miR-1245	Down	–1.66	1.65E-04
GSE61741	miRNA	hsa-miR-155*	Down	–1.60	3.32E-04
GSE24519	mRNA	ATXN7L1	Up	11.32	3.71E-08
GSE24519	mRNA	PURA	Up	10.78	7.69E-32
GSE24519	mRNA	KRI1	Up	10.63	1.23E-09
GSE24519	mRNA	C2orf48	Up	10.51	8.58E-06
GSE24519	mRNA	HNRNPA2B1	Up	10.00	1.98E-13
GSE24519	mRNA	LALBA	Down	–5.73	2.04E-06
GSE24519	mRNA	TBX19	Down	–5.14	1.24E-02
GSE24519	mRNA	SRI	Down	–4.58	2.49E-04
GSE24519	mRNA	P3H2	Down	–4.57	2.93E-04
GSE24519	mRNA	MARS2	Down	–4.25	2.34E-05

### DEcircRNA Annotation and Structure Prediction

**FIGURE 3:**
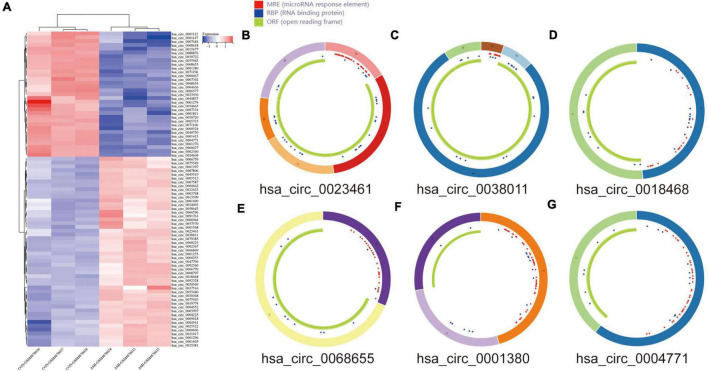
DEcircRNAs in GES160717. **(A)** Heatmap of DEcircRNAs in GSE160717. **(B)** Structural pattern of hsa_circ_0023461. **(C)** Structural pattern of hsa_circ_0038011. **(D)** Structural pattern of hsa_circ_0018468. **(E)** Structural pattern of hsa_circ_0068655. **(F)** Structural pattern of hsa_circ_0001380. **(G)** Structural pattern of hsa_circ_0004771.

The DEcircRNA’s name in GSE160717 containing six digits was converted into circBase ID containing seven digits (Supplementary File 2). Figure 3A is the Heatmap of these DEcircRNAs. DEcircRNAs were entered into the circBase database to get their annotation information. The annotation information of the top 5 high-expressed DEcircRNAs and the top 5 low-expressed DEcircRNAs are shown in Table 3. Structure of these DEcircRNAs were predicted and visualized using CSCD database. The structural patterns of the top 3 high-expressed DEcircRNAs and the top 3 low-expressed DEcircRNAs are shown in Figures 3B–G.

**TABLE 3:** The annotation information of the top 5 high-expressed DEcircRNAs and the top 5 low-expressed DEcircRNAs.

circRNA name	circRNA circBase ID	Position	Genomic length	Strand	Gene symbol	Expression
hsa_circRNA_023461	hsa_circ_0023461	chr11:72406762-72423384	16622	–	ARAP1	Up
hsa_circRNA_038011	hsa_circ_0038011	chr16:15120499-15123913	3414	+	PDXDC1	Up
hsa_circRNA_400564	hsa_circ_0018468	chr10:65326097-65354614	28517	+	REEP3	Up
hsa_circRNA_400027	hsa_circ_0092367	chr15:25325262-25326442	1180	+	SNORD116-14	Up
hsa_circRNA_400101	hsa_circ_0092328	chr9:136216037-136216257	220	+	SNORD24	Up
hsa_circRNA_068655	hsa_circ_0068655	chr3:196129822-196134264	4442	–	UBXN7	Down
hsa_circRNA_103560	hsa_circ_0001380	chr3:196118683-196129890	11207	–	UBXN7	Down
hsa_circRNA_103110	hsa_circ_0004771	chr21:16386664-16415895	29231	–	NRIP1	Down
hsa_circRNA_015677	hsa_circ_0015677	chr1:185153374-185200840	47466	+	SWT1	Down
hsa_circRNA_103111	hsa_circ_0061276	chr21:16415815-16415895	80	–	NRIP1	Down

### Construction of the circRNA-miRNA-mRNA Regulatory Network

**FIGURE 4:**
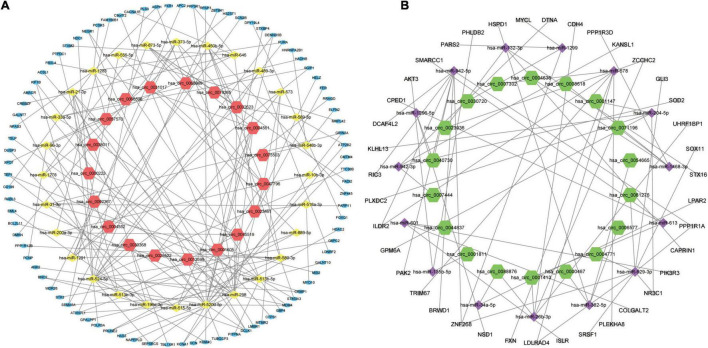
circRNA-miRNA-mRNA regulatory Network of AMI. **(A)** Up-regulated circRNA corresponding circRNA-miRNA-mRNA regulatory Network. **(B)** Down-regulated circRNA corresponding circRNA-miRNA-mRNA regulatory Network. For panels **(A,B)**, the orange-red regular hexagon represents up-regulated circRNA, the green regular hexagon represents down-regulated circRNA, the yellow diamond represents down-regulated miRNA, the purple diamond represents up-regulated miRNA, the blue oval represents up-regulated mRNA and the gray oval represents down-regulated mRNA.

In order to better understand the molecular mechanism of circRNAs involved in the process of AMI. The up-regulated circRNAs and down-regulated circRNAs were used to construct a circRNA-miRNA-mRNA regulatory network respectively. Figure 4A is an “up-regulated circRNA”-“down-regulated miRNA”-“up-regulated mRNA” network, with 134 nodes and 166 edges, including 19 up-regulated circRNAs, 28 down-regulated miRNA and 87 up-regulated mRNAs. On the contrary, Figure 4B is a “down-regulated circRNA”-“up-regulated miRNA”-“down-regulated mRNA” network, with 72 nodes and 68 edges, including 18 down-regulated circRNAs, 15 up-regulated miRNA and 39 down-regulated mRNAs. These RNA interactions may provide new insight into the mechanism underlying AMI.

### Analysis of Enrichment of Genes in the circRNA-miRNA-mRNA Regulatory Network

**FIGURE 5:**
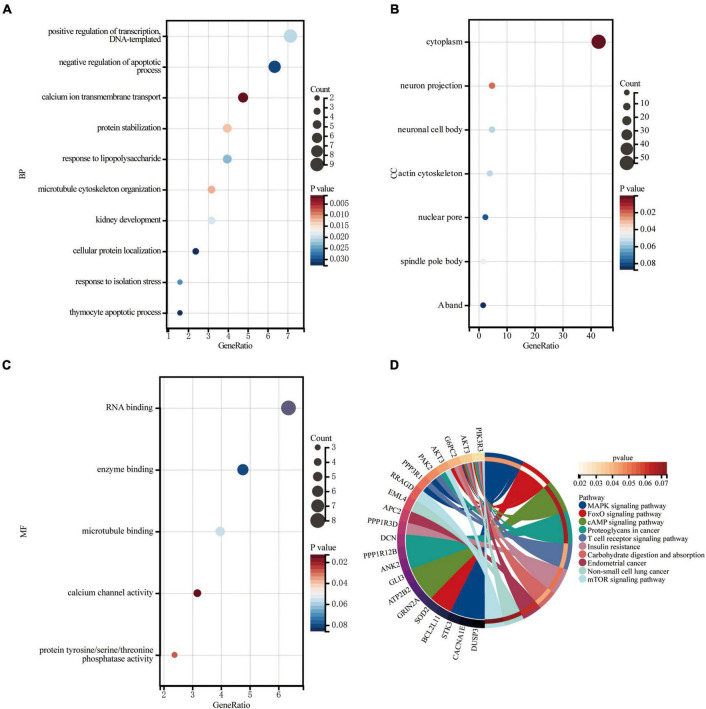
Enrichment of genes in the circRNA-miRNA-mRNA Regulatory Network. **(A)** Bubble plot of biological process (BP; TOP10). **(B)** Bubble plot of cellular component (CC; TOP6). **(C)** Bubble plot of molecular function (MF; TOP5). **(D)** Circle plot of KEGG pathway enrichment analysis (TOP10).

Gene Ontology knowledgebase and KEGG database were used to characterize the functional roles of the DEGs in the circRNA-miRNA-mRNA Regulatory Network mentioned above. Figure 5 list the top10 enriched GO terms and KEGG pathways. The biological process (BP) category of the GO analysis results showed that these DEGs are significantly enriched in the term of “positive regulation of transcription, DNA-templated,” “negative regulation of apoptotic process,” and “calcium ion transmembrane transport,” etc. (Figure 5A). For GO cellular component (CC) analysis, the top 4 significantly enriched terms are “cytoplasm,” “neuron projection,” “neuronal cell body,” “actin cytoskeleton,” (Figure 5B). The top four significantly enriched molecular function (MF) terms include “RNA binding,” “enzyme binding,” “microtubule binding,” and “calcium channel activity” (Figure 5C). Furthermore, “FOXO signaling pathway,” “Carbohydrate digestion and absorption,” “T cell receptor signaling pathway,” “MAPK signaling pathway,” “Insulin resistance,” “cAMP signaling pathway,” and “mTOR signaling pathway” are pathways of significant enrichment in KEGG analysis (Figure 5D).

### Construction of the Protein-Protein Interaction Network and Identification of Hub Genes and Key Modules

**FIGURE 6:**
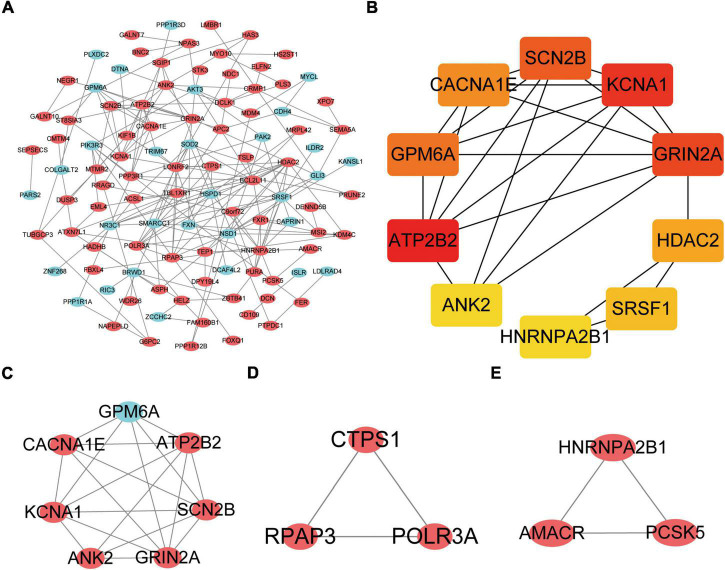
Protein–Protein Interaction Network, Hub Genes and Key Modules. **(A)** The whole PPI Network. The 74 orange red nodes in the network represent up-regulated genes and the 31blue nodes in the network represent down-regulated genes. **(B)** PPI network of the 10 hub genes. **(C)** PPI network of module 1. **(D)** PPI network of module 2. **(E)** PPI network of module 3.

The DEGs obtained from the circRNA-miRNA-mRNA Regulatory Network were introduced into the online database String. After removing the isolated genes without interaction, a PPI network with 105 nodes and 203 edges was established (Figure 6A). The 74 orange red nodes in the network represent up-regulated genes and the 31 blue nodes in the network represent down-regulated genes. Next, based on MCC method, the top 10 hub genes were filtered out using the plug-in Cytohubba in Cytoscape. They were ATP2B2, KCNA1, GRIN2A, SCN2B, GPM6A, CACNA1E, HDAC2, SRSF1, ANK2, and HNRNPA2B1. The PPI network of these 10 hub genes is shown in Figure 6B. On the basis of MCODE, we identified 3 modules in the whole network (Figures 6C–E).

The KOBAS online tool was used for pathway enrichment analysis. The results indicated that “the DEGs in module 1” and “hub genes” are significantly enriched in the pathway of Calcium signaling pathway. The DEGs of module 2 are enriched in the pathway of RNA polymerase. In addition, the DEGs of module 3 are significantly enriched in the pathway of Peroxisome. Table 4 shows the top three significant modules and hub genes in the PPI network.

**TABLE 4:** The top three significant modules and hub genes in the PPI network.

Plug-in	Modules	Nodes	Edges	Genes	Pathway	*P*-value
MCODE	Module 1	7	19	ATP2B2, KCNA1, GRIN2A, SCN2B, GPM6A CACNA1E, ANK2	Calcium signaling pathway	1.01E-04
MCODE	Module 2	3	3	CTPS1, RPAP3, POLR3A	RNA polymerase	6.51E-03
MCODE	Module 3	3	3	AMACR, HNRNPA2B1, PCSK5	Peroxisome	9.61E-03
CytoHubba	Hub gene	10	23	ATP2B2, KCNA1, GRIN2A, SCN2B, GPM6A CACNA1E, HDAC2, SRSF1, ANK2,HNRNPA2B1	Calcium signaling pathway	5.45E-04
						

### Hub Gene Related circRNAs AND miRNAs

**FIGURE 7:**
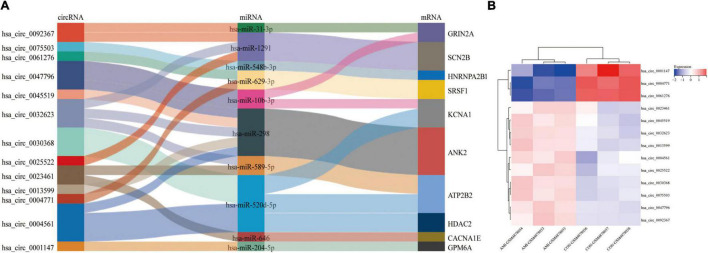
Hub gene Related circRNAs and miRNAs. **(A)** The sankey diagram of the circRNA-miRNA-Hub gene network. **(B)** Heatmap of hub gene-related circRNAs.

Figure 7A is the sankey diagram of hub genes, hub gene related miRNAs and circRNAs selected from the circRNA-miRNA-mRNA regulatory network mentioned above. This sankey diagram contains 13 circRNAs,10 miRNAs and 10 hub genes, as well as 24 competing endogenous regulatory relationships. For example, hsa_circ_0023461 might act as a hsa-miR-589-5p sponge and play a regulatory role in acute myocardial infarction by influencing the expression of ATP2B2. In order to further analyze the relative expression of circRNA in this sankey diagram, heat map was used to further demonstrate (Figure 7B).

### Identification and Validation of Potential Biomarkers in Datasets

**FIGURE 8:**
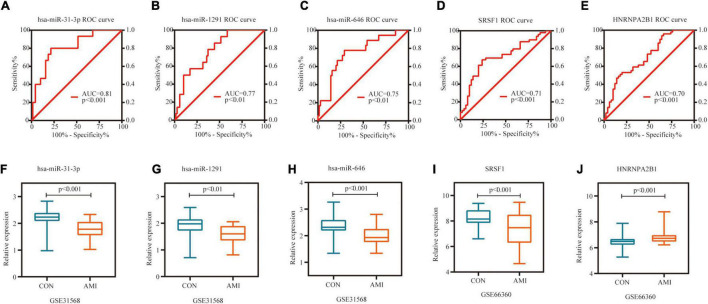
Potential biomarkers in datasets. **(A)** ROC curve of hsa-miR-31-3p in GSE31568. **(B)** ROC curve of hsa-miR-1291 in GSE31568. **(C)** ROC curve of hsa-miR-646 in GSE31568. **(D)** ROC curve of SRSF1 in GSE66360. **(E)** ROC curve of HNRNPA2B1 in GSE66360. **(F)** The relative expression of hsa-miR-31-3p in GSE31568. **(G)** The relative expression of hsa-miR-1291 in GSE31568. **(H)** The relative expression of hsa-miR-646 in GSE31568. **(I)** The relative expression of SRSF1 in GSE66360. **(J)** The relative expression of HNRNPA2B1 in GSE66360.

Receiver operating characteristic curve (ROC) analysis was performed on 10 miRNAs and 10 hub genes in the Sankey diagram and AUC values were calculated. Meanwhile, molecules with AUC ≥ 7 and *P* < 0.05 in two datasets were considered as potential biomarkers. Finally, hsa-miR-31-3p, hsa-miR-1291, hsa-miR-646, SRSF1 and HNRNPA2B1 were identified as potential markers of AMI. Figures 8A–E shows the detailed results of ROC analysis. GSE31568 (miRNA) and GSE66360 (mRNA) were used to further evaluate the relative expression of these molecules. As predicted by the previous analysis, hsa-miR-31-3p, hsa-miR-1291, hsa-miR-646, and SRSF1 were decreased, while HNRNPA2B1 was increased (Figures 8F–J).

### qRT-PCR Verification of Hub Genes and Related circRNAs

**FIGURE 9:**
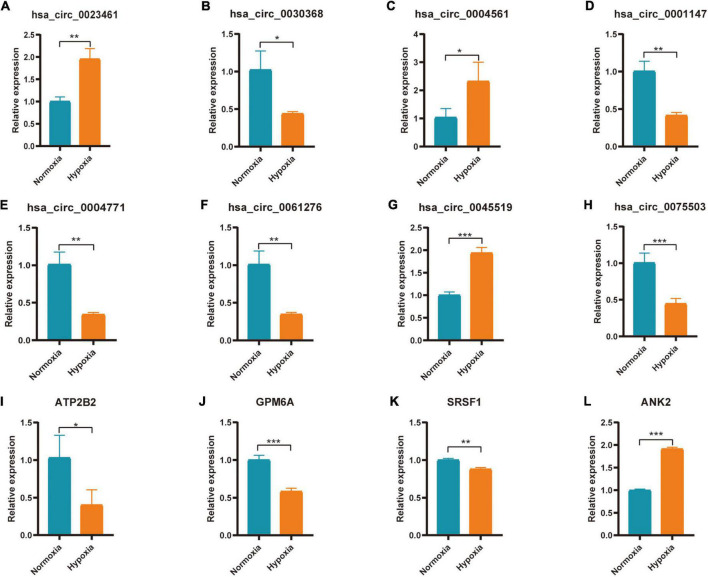
Expression of Hub gene and Hub gene related circRNAs in AC16 cell hypoxia model. For panels **(A–L)** *P < 0.05; **P < 0.01; ***P < 0.001.

Hub genes (ATP2B2, KCNA1, GRIN2A, SCN2B, GPM6A, CACNA1E, HDAC2, SRSF1, ANK2, and HNRNPA2B1) and Hub gene related circRNAs (hsa_circ_0023461, hsa_circ_0032623, hsa_circ_0030368, hsa_circ_0004561, hsa_circ_0047796, hsa_circ_0092367, hsa_circ_0025522, hsa_circ_0001147, hsa_circ_0004771, hsa_circ_0061276, hsa_circ_0013599, hsa_circ_0045519, hsa_circ_0075503)were further verified in AC16 hypoxia modle. The results showed that 4 Hub genes and 8 Hub gene related circRNAs were differentially expressed. They were hsa_circ_0023461(up), hsa_circ_0030368 (down), hsa_circ_0004561 (up), hsa_circ_0001147 (down), hsa_circ_0004771(down), hsa_circ_0061276 (down), hsa_circ_0045519(up), hsa_circ_0075503(down), ATP2B2(down), GPM6A (down), SRSF1(down), and ANK2(up) (Figures 9A–L). The expression of hsa_circ_0023461, hsa_circ_0004561, hsa_circ_0001147, hsa_circ_0004771, hsa_circ_0061276, hsa_circ_0045519, GPM6A, SRSF1, and ANK2 werre consistent with the predicted result. Therefore, there may be hsa_circ_0001147/hsa-miR-204-5p/GPM6A axis, hsa_circ_0004771/hsa-miR-629-3p/SRSF1 axis, hsa_circ_0061276/hsa-miR-629-3p/SRSF1 axis, hsa_circ_0045519/hsa-miR-298/ANK2 axis, and hsa_circ_0004561 hsa-miR-298/ANK2 axis regulatory relationship involved in myocardial infarction.

## Discussion

Acute myocardial infarction has a high incidence and mortality in the middle-aged and elderly worldwide. In the AMI-related research field, circRNA, as a key molecule for gene expression regulation, has attracted more and more attention from scientists in recent years. However, the available circRNA information of AMI is still very limited, and the selection of AMI-related key circRNAs is also a challenge. We use bioinformatics method to conduct data mining on AMI-related datasets, extract relevant biological information from high-dimensional data, and construct the corresponding circRNA-miRNA-mRNA regulatory Network, aiming to explore the mechanism of circRNA involvement in acute myocardial infarction. These identified circRNA and circRNA-related molecules may be used as biomarkers for AMI, providing basis for further scientific research and individualized diagnosis and treatment of clinical patients.

In the present study, we screened 83 DEcircRNAs, 109 CoDEmiRNAs and 1204 DEGs in AMI-related datasets. Subsequently, the endogenous competition regulatory networks corresponding to the up-regulated and down-regulated circRNAs were constructed, respectively. GO and KEGG pathway enrichment analysis showed that DEGs in these two networks were significantly enriched in pathways related to cell cycle, apoptosis, autophagy and immune. These circRNA-related DEGs are involved in “FOXO signaling pathway,” “T cell receptor signaling pathway,” “MAPK signaling pathway,” “Insulin resistance,” “cAMP signaling pathway,” and “mTOR signaling pathway.” These discovered pathways may be the key molecular mechanism for circRNAs participating in AMI. Based on PPI analysis, 10 hub genes, ATP2B2, KCNA1, GRIN2A, SCN2B, GPM6A, CACNA1E, HDAC2, SRSF1, ANK2, and HNRNPA2B1 were identified. These genes are enriched in the pathway of Calcium signaling pathway. This indicated that circRNA may also participate in the regulation of calcium signaling pathway. In addition, 10 miRNAs and 13 circRNAs related to these 10 hub genes were screened out and hsa-miR-31-3p, hsa-miR-1291, hsa-miR-646, SRSF1 and HNRNPA2B1 were identified as potential markers of AMI.

CircRNA is stable and highly conserved among species, so it may be a key marker or therapeutic target for disease (33, 34). A diagnostic experimental study, including 1842 patients and 316 controls, showed that circ-YOD1 may act as a new biomarker for CAD (AUC = 0.824) (35). Our previous research also shows that CircSAMD4A can sponge miR-138-5p to promote H/R-induced inflammatory response and apoptosis (36). Therefore, circRNA may be involved in the process of AMI, and the circRNAs identified by us such as, hsa_circ_0023461, hsa_circ_0004561, hsa_circ_0001147, hsa_circ_0004771, hsa_circ_0061276 and hsa_circ_0045519, etc, are likely to be potential markers or therapeutic targets of AMI. Further studies are needed to verify these circRNAs.

Some circRNA-related molecules such as hsa-miR-31-3p, hsa-miR-1291, hsa-miR-646, SRSF1 and HNRNPA2B1 have also been identified. One study has shown that miR-31 promotes poor cardiac remodeling and dysfunction in ischemic heart disease and therapeutic inhibition *in vivo* can improve cardiac insufficiency and prevent the development of poor remodeling (37). Another diagnostic experimental study, including 80 AMI patients and 80 controls, showed that miR-1291 may act as a new biomarker for AMI (38). SRSF1 (serine and arginine rich splicing factor 1) encodes a member of the arginine/serine-rich splicing factor protein family, which plays an important role in the regulation of gene expression in the immune system (39). HNRNPA2B1 (heterogeneous nuclear ribonucleoprotein A2/B1) is a kind of RNA binding protein, which complex with heterogeneous nuclear RNA (hnRNA) (40). One study showed that downregulation of HNRNPA2B1 reduced proliferation of vascular smooth muscle (41). As for the role of these molecules in AMI, further research is needed to prove them.

Further qPCR verification indicated the mechanism of circRNA participating in hypoxia-induced myocardial injury. There may be hsa_circ_0001147/hsa-miR-204-5p/GPM6A axis, hsa_circ_0004771/hsa-miR-629-3p/SRSF1 axis, hsa_circ_0061276/hsa-miR-629-3p/SRSF1 axis, hsa_circ_ 0045519/hsa-miR-298/ANK2 axis and hsa_circ_0004561 hsa-miR-298/ANK2 axis regulatory relationship involved in myocardial infarction. Further experimental verification is needed.

The above research results are comprehensive analysis of existing datasets. Therefore, these conclusions lack enough objective experimental evidence to support them, so they can only be the preliminary part of further research. In the future, we will carry out research work in large samples of people, and at the same time, we will further study the molecular mechanism of circRNA involved AMI in animal model.

## Conclusion

In conclusion, we identified 83 DEcircRNAs and constructed the corresponding circRNA-miRNA-mRNA regulatory network. Bioinformatics analyses showed 43 miRNAs and 126DEGs were associated with these circRNA and they affect AMI through “FOXO signaling pathway,” “T cell receptor signaling pathway,” “MAPK signaling pathway,” “Insulin resistance,” “cAMP signaling pathway,” and “mTOR signaling pathway.” CircRNA-related molecules such as hsa_circ_0023461, hsa_circ_0004561, hsa_circ_0001147, hsa_circ_0004771, hsa_circ_0061276, hsa_circ_0045519, GPM6A, SRSF1 and ANK2 may be potential biomarkers of AMI.

## Data Availability Statement

The datasets presented in this study can be found in online repositories. The names of the repository/repositories and accession number(s) can be found in the article/Supplementary Material.

## Author Contributions

JW, CL, and ZL performed the data analysis and drafted the manuscript. HC, YH, and YZ prepared the figures and contributed toward the study design. TZ and HZ optimized the analysis protocol and participated in the data analysis. XH and JC revised the figures and designed and supervised the study. All authors have read and approved the final manuscript.

## Conflict of Interest

The authors declare that the research was conducted in the absence of any commercial or financial relationships that could be construed as a potential conflict of interest.

## Publisher’s Note

All claims expressed in this article are solely those of the authors and do not necessarily represent those of their affiliated organizations, or those of the publisher, the editors and the reviewers. Any product that may be evaluated in this article, or claim that may be made by its manufacturer, is not guaranteed or endorsed by the publisher.

## Footnotes

1
https://www.ncbi.nlm.nih.gov/

2
https://www.ncbi.nlm.nih.gov/geo/geo2r/

3
http://bioinformatics.psb.ugent.be/webtools/Venn/

4
http://circrna.org

5
http://mirdb.org

6
http://gb.whu.edu.cn/CSCD

7
http://www.circbank.cn/searchmiRNA.html

8
https://circinteractome.nia.nih.gov/

9
http://mirdb.org

10
http://targetscan.org

11
http://mirwalk.umm.uni-heidelberg.de/

12
https://david.ncifcrf.gov

13
http://string-db.org

14
http://kobas.cbi.pku.edu.cn/

## Abbreviations

AMI: acute myocardial infarction
circRNA: circular RNA
lncRNA: long non-coding RNA
miRNA: microRNA
ncRNA: non-coding RNA
GEO: Gene Expression Omnibus
DERNA: Differential Expression RNA
PPI: protein–protein interaction
DEG: differentially expressed gene
PCI: percutaneous coronary intervention
CFs: cardiac fibroblasts
MRE: microRNA response element
RBP: RNA binding protein
ORF: open reading frames
GO: Gene Ontology
KEGG: Kyoto Encyclopedia of Genes and Genomes
BP: biological process
CC: cellular component
MF: molecular function
ROC: receiver operating characteristic curve.
